# Reminiscence and grief resolution through online journalling: mixed-methods analysis of the Living Memory Home bereavement cohort

**DOI:** 10.1192/bjo.2025.10882

**Published:** 2025-10-28

**Authors:** Courtney E. Lee, Holly G. Prigerson, Francesca Falzarano, Madeline Rogers, Francesco Osso, Sosi Korian, Charlotte LaPlante, Madison Pavao, Jin Pyon, Wan Jou She, Hillary Winoker, Alexandra Pitman, Kailey Roberts, Paul K. Maciejewski

**Affiliations:** Weill Cornell Medicine, New York, NY, USA; Cornell Center for Research on End-of-Life Care, New York, NY, USA; Department of Radiology, Weill Cornell Medicine, New York, NY, USA; Leonard Davis School of Gerontology, University of Southern California, Los Angeles, CA, USA; Department of Psychiatry, Brigham and Women’s Hospital, Boston, MA, USA; Department of Information and Human Sciences, Kyoto Institute of Technology, Japan; Division of Psychiatry, University College London, UK; North London NHS Foundation Trust, London, UK; Ferkauf Graduate School of Psychology, Yeshiva University, New York, NY, USA

**Keywords:** Prolonged grief disorder, Prolonged Grief Disorder-Revised scale (PG-13-R), reminiscence, online bereavement support, mixed methods

## Abstract

**Background:**

Prolonged grief disorder (PGD) is a chronic, impairing state of intense grief that is responsive to specialised intervention. Online journalling has the potential to reduce PGD symptoms.

**Aims:**

To assess whether reminiscence via online journalling facilitates bereavement adjustment – specifically, to identify key themes in online grief journals and examine associations between types of reminiscence and changes in PGD symptom severity.

**Method:**

A cohort of 96 bereaved adults completed 7 days of online journalling on the Living Memory Home (LMH) bereavement website. Participants were recruited from clinics, bereavement support groups and a National Institutes of Health-funded, web-based platform. The Prolonged Grief Disorder-Revised scale (PG-13-R) was administered at baseline and at 1-week and 1-month follow-up.

**Results:**

Descriptive analysis revealed a reduction in PG-13-R scores from baseline to 1-week follow-up (mean difference −3.7, 95% CI: −4.9, −2.5, *P* < 0.001) and 1-month follow-up (mean difference −5.0, 95% CI: −6.4, −3.7. *P* < 0.001). Mixed-methods analysis revealed significant negative associations between reflection on negative traits of the deceased and PG-13-R score at all three time points, and between reflection on past experiences with the deceased and PG-13-R score at baseline. Expression of regret and guilt was significantly associated with reduction in PG-13-R scores from 1-week to 1-month follow-up.

**Conclusions:**

Engagement in the LMH website demonstrated significant declines in PGD symptom severity after 1 week of online journalling and at 1-month follow-up. Guided reflection on memories of the deceased, and even on negative or emotionally challenging memories, shows potential for reducing symptoms of PGD.

Reminiscence, or the recollection of memories, is characteristic of mourning. In an adaptive mourning process, the bereaved person maintains connection with the deceased through reminiscence and, over time, integrates the reality of the loss with existing memories.^1^ Reminiscence is thus a topic of potential relevance to the design of interventions aimed at reducing symptoms of prolonged grief disorder (PGD), a chronically intense, impairing state of distressing grief.^2,3^

## Background studies

Research shows that avoiding reminders and memories of the deceased is associated with poor bereavement outcomes.^4–6^ In addition, grief therapies that target cognitive reminder avoidance and encourage reminiscence of memories of the deceased person have been found to be more effective than supportive counselling, mindfulness-based cognitive therapy and antidepressant monotherapy in improving grief outcomes.^7–10^ Nonetheless, not all types of reminiscence may facilitate bereavement adjustment.

Reminiscence can be categorised as either functionally reflective – motivated by identity construction and problem-solving, or ruminative – motivated by bitterness revival and boredom reduction.^11,12^ Grief rumination is further characterised by negative focus on challenging emotions in grief (e.g. bitterness, regret, guilt), thoughts on the injustice or consequences of the loss and counterfactual thinking (i.e. dwelling on alternative scenarios such as preventing the death).^13^ Although both reflection and rumination engage with past-oriented thoughts, rumination is associated with poor adaptation to grief – manifesting as preoccupation, difficulty establishing trust relationships and excessive distress.^5,14,15^ It has thus been hypothesised that rumination is an avoidant coping style that provides a defensive escape from confronting the painful reality of the loss. Rumination is thought to hinder the direct reflection and processing of salient memories, an essential element of ‘grief work’ – mental processing of the loss.^16–18^ In contrast, reflection that functions as meaning reconstruction – the process of making sense of the loss and reconfiguring new relationships with the deceased, others and oneself – is associated with better bereavement outcomes.^14,19–21^

While the relationship between reminiscence functions and bereavement adjustment has been well described,^11,12,14,19–21^ studies have not examined, on a more granular level, whether the content or emotional valence of reminiscence is related to bereavement outcomes. It remains unclear, for example, whether reflection on negative, distressing memories is linked to worse outcomes than reflection on mostly positive ones. Here, we posit that what matters for adaptive mourning is not necessarily the emotional valence of memories but rather the act of engaging with memories – good or bad.

## Aims

We aimed to examine the relationship between reminiscence and bereavement adjustment using free-text data from the Living Memory Home (LMH) prospective cohort study. The LMH is a digital platform hosted on the Cornell Center for Research on End-of-Life Care website for bereaved individuals as a resource for memorialising deceased family members and friends.^22^ With the increasingly common usage of digital media (digital photographs, videos, blogs) to maintain connections with deceased loved ones,^23,24^ the LMH was designed as a controlled, accessible website for bereaved individuals to maintain bonds and express emotions. This study aimed to assess associations between online journal writing and bereavement adjustment (i.e. PGD symptom severity over time). A mixed-methods approach was used to identify key themes in LMH journal entries, and to explore associations between types of reminiscence – thematically coded by content and emotional valence – and changes in PGD symptom severity before and after LMH engagement.

## Method

### Recruitment

Adults living in New York State and bereaved by the death of a close person (e.g. family member, friend) were recruited through flyers at bereavement support groups and relevant clinics and websites at Weill Cornell Medicine (e.g. Wright Center on Aging, Department of Geriatrics and Palliative Medicine, cancer clinic bulletin boards, Acute Care for the Elderly Unit, the Medical Intensive Care Unit, Cornell Center for Research on End-of-Life Care website), by direct mailing to people referred from these clinics and via advertisements on ResearchMatch.org – an online platform sponsored by the National Institutes of Health (NIH) to connect people with NIH-funded research studies. Potential participants completed the validated Prolonged Grief Disorder-Revised (PG-13-R) grief severity scale,^2^ Columbia Suicide Severity Rating Scale^25^ and an established sociodemographic and loss characteristics questionnaire.^26^ Exclusion criteria included not having internet access or knowledge of how to operate a computer or smartphone; not being fluent in English; belonging to a specific vulnerable population (prisoners, pregnant persons, children); and endorsement of suicide attempt or suicidal ideation with plan and/or intent within the past 12 months (for risk management reasons). Verbal consent was witnessed via telephone and formally recorded by a research staff member.

### Study procedure

Eligible participants were given a secure personal account and a brief tutorial on using the LMH website. The LMH journal writing feature offered a selection of 44 open-ended writing prompts that encouraged reflection, expression of grief-related thoughts and feelings and imagined dialogues with the deceased, expanded from the therapeutic prompts by Neimeyer’s ‘Correspondence with the Deceased’ in *Techniques in Grief Therapy.* ^27^ Writing prompts elicited specific memories (e.g. ‘One of my most treasured memories of you is …’) and emotions in grief (e.g. ‘Now that you’re gone I feel …’). The full list of writing prompts is provided in Supplementary Appendix 2 available at https://doi.org/10.1192/bjo.2025.10882. Participants completed 7 consecutive days of journal writing in the LMH for at least 20 min per day. Participants chose writing prompts at will, and there was no limit on the length or number of entries that could be entered. Data were collected from 29 April 2021 to 30 April 2023.

### Ethical standards

The authors assert that all procedures contributing to this work comply with the ethical standards of the relevant national and institutional committees on human experimentation, and with the Helsinki Declaration of 1975 as revised in 2013. All procedures involving human subjects/patients were approved by the Weill Cornell Medicine Institutional Review Board (Approval no. 1810019629).

### Measures

Participants self-reported sociodemographic and loss-related characteristics. PGD symptom severity was measured by PG-13-R score at baseline (prior to LMH login), at 1 week (on completion of the 7-day LMH journal writing protocol) and at 1 month (30 days after the first recorded journal entry). The PG-13-R scale ranges from 10 to 50, with scores of 30 or above indicating a clinically significant threshold of grief severity that may serve as a diagnostic criterion for PGD.^2^ We used change in this score to capture bereavement adjustment.

### Thematic analysis

We used thematic analysis to capture key themes and codes in free-text data from LMH journal entries, for use as variables in statistical analysis. Details regarding data extraction, coding team, codebook development, coding protocol, final coding and code frequency are provided in Supplementary Appendix 1.

### Derived variables of reminiscence

A team approach was used to derive variables based on codes from the thematic analysis, used as proxies for key constructs in relation to the process of reminiscence. From the codebook we identified codes that captured reflective reminiscence and grief rumination.

### Statistical analysis

We quantified reminiscence variables by their code frequency, the total number of text entries expressing that code and participant. Spearman partial correlation coefficients were used to estimate associations between code frequency and two sets of outcomes: (a) PG-13-R scores at baseline, 1 week and 1 month, (b) and change in PG-13-R scores from baseline to 1 week, baseline to 1 month and 1 week to 1 month. Correlation coefficients controlled for the total number of journal entries per participant over the course of LMH use. We identified potential confounders by estimating correlations between participant characteristics and outcomes (PG-13-R scores and code frequencies). Four participants had missing or incomplete PG-13-R assessments; for missing values we imputed their mean values.

## Results

### Participant characteristics

Of 137 participants, 96 completed the study, 41 withdrew or were lost to follow-up and none were excluded. Our analysis included 2390 journal entries from 96 participants. Characteristics of study completers are summarised in Table 1. The mean age was 44.5 years (s.d. = 16.5), ranging from 20 to 89 years. Regarding demographic factors, 77 (80.2%) were female, 73 (76.8%) were heterosexual, 71 (74.0%) were White, 53 (55.2%) had attained a graduate or professional degree and 52 (54.2%) lived in New York City. Regarding loss-related characteristics, 42 (43.8%) had lost a parent, 15 (15.6%) a grandparent, 14 (14.6%) a spouse or partner, 7 (7.3%) a child, 4 (4.2%) a sibling and 11 (11.5%) a friend or other non-family other. Mean time since loss was 58.8 months (s.d. = 85.7), with a median of 24 months, ranging from 2 months to 38 years. In 55 (57.3%) cases the death was rated by the participant as unexpected, including causes of natural sudden death (e.g. heart attack), COVID-19, homicide, suicide, accidental overdose, fatal accident (e.g. car accident), natural disaster (e.g. earthquake) and others.

**Table 1:** Sample demographic and loss-related characteristics

Demographic or loss-related characteristic^a^	*n* (%) (*N* = 96)
Age (years), mean (s.d.) [range]	44.5 (16.5) [20–89]
Gender^b^	
Male	18 (19.0)
Female	77 (80.2)
Sexual orientation^c^	
Heterosexual	73 (76.8)
Lesbian	6 (6.3)
Gay	4 (4.2)
Bisexual	3 (3.2)
Queer	7 (7.4)
Other	2 (2.1)
Race	
American Indian or Alaska Native	1 (1.0)
Asian	7 (7.3)
African American or Black	12 (12.5)
White	71 (74.0)
Other	5 (5.2)
Ethnicity	
Hispanic, Latino or of Spanish origin	7 (7.3)
Not Hispanic, Latino or of Spanish origin	89 (92.7)
Highest education level	
High school	8 (8.3)
College	35 (36.5)
Graduate/professional	53 (55.2)
Location	
New York City (NYC)	52 (54.2)
New York State (outside of NYC’s five boroughs)	44 (45.8)
Time since loss (months),^d^ mean (s.d.) [range]	58.8 (85.7) [2–456]
Relationship of deceased to participant	
Spouse/partner	14 (14.6)
Child	7 (7.3)
Parent	42 (43.8)
Grandparent	15 (15.6)
Sibling	4 (4.2)
Friend	3 (3.1)
Other	11 (11.5)
Cause of death	
Natural anticipated death (e.g. terminal illness)	41 (42.7)
Natural sudden death (e.g. heart attack)	22 (22.9)
COVID-19	5 (5.2)
Homicide	1 (1.0)
Suicide	4 (4.2)
Accidental overdose	2 (2.1)
Fatal accident (e.g. car accident)	7 (7.3)
Natural disaster (e.g. earthquake)	1 (1.0)
Other	13 (13.5)

a. Sample characteristics were self-reported.

b. Missing data from one participant.

c. Missing data from one participant.

d. Missing data from 14 participants.

We collected sociodemographic data for 36 of the 41 participants who withdrew or were lost to follow-up. This group had a mean age of 53.7 years (s.d. = 18.1). Of those 36 participants who withdrew or were lost to follow-up, 27 (75.0%) were female, 32 (88.9%) were heterosexual, 17 (47.2%) had attained a graduate or professional degree and 19 (52.8%) lived in New York City. Regarding race and ethnicity, 21 (58.3%) were White, 9 (25.0%) were African American or Black and 7 (19.4%) were Hispanic/Latino. Regarding loss-related characteristics, 13 (36.1%) had lost a parent, 2 (5.6%) a grandparent, 5 (13.9%) a spouse or partner, 4 (11.1%) a child, 3 (8.3%) a sibling and 9 (25.0%) a friend or other non-family other. In 18 (50.0%) cases, the death was rated by the participant as unexpected.

### Thematic analysis

We identified 22 codes (subthemes) and three main themes: (a) reflection, (b) continued bonds and (c) psychosocial adjustment. Reflection captured reminiscence on memories of the deceased, as well as present- and future-oriented musings. Continued bonds described ways of maintaining a connection with the deceased, such as imagining dialogues, emulating the traits of the deceased or memorialising the deceased through significant objects, places or actions. Psychosocial adjustment captured emotional reactions to the loss, coping mechanisms and changes in one’s life and relationships following the loss. Organisation, definitions and textual examples of all codes are provided in Supplementary Table 1.

### Derived variables of reminiscence

We identified five codes as conceptually meaningful variables for examination of reminiscence in the LMH journals: experiences with the deceased, traits of the deceased, death circumstances, bitterness, and regret and guilt. Code frequencies are provided in Supplementary Table 2.

Experiences, traits and death circumstances were selected as measures of reflective reminiscence: past-oriented reflections on specific memories of the deceased and the loss event. Experiences captured memories of experiences with the deceased, further categorised by emotional valence. Positive, negative and neutral/unclear experiences appeared in 338, 129 and 51 entries, respectively, out of the total 2390 entries. Traits captured reflection on specific characteristics of the deceased, further categorised by positive or negative emotional valence. Positive traits appeared more frequently than negative, in 541 and 134 entries, respectively. We did not identify traits with neutral/unclear valence. Death circumstances, coded in 241 entries, captured reflection on the death event and the circumstances preceding the death (e.g. a hospital course leading up to the death) or following the death (e.g. making funeral arrangements).

Bitterness and regret and guilt were selected to explore the negative emotional reactions associated with grief rumination.^13^ Bitterness captured negative emotions towards the deceased, other people or one’s circumstances, often using exaggerated language to express anger, disappointment or resentment. Regret and guilt captured repentance, remorse or the wish that different actions had been taken in the past. Often, regret and guilt coincided with expressions of pity, empathy and ways in which the bereaved individual would have changed their behaviour in hindsight (e.g. wishing they had spent more time with the deceased before the death). Bitterness appeared in 159 entries, regret and guilt in 188.

### Changes in PGD symptom severity

Table 2 shows the main outcomes of PGD symptom severity: mean PG-13-R scores at baseline, 1 week and 1 month and mean changes in PG-13-R scores from baseline to 1 week and 1 month. At baseline, PG-13-R scores were below the PGD diagnostic threshold of 30 for 84 (87.5%) of the 96 participants, with an overall mean score of 24.9 (s.d. = 8.2) at baseline, 21.2 (7.0) at 1-week and 19.8 (6.8) at 1-month follow-up. Results revealed significant reductions in PG-13-R scores from baseline values at 1-week (mean difference [s.d.], −3.7 [6.0], *P* < 0.001) and at 1-month follow-up (mean difference [s.d.], −5.0 [6.9], *P* < 0.001).

**Table 2:** Grief severity (Prolonged Grief Disorder-Revised scale, PG-13-R) at baseline, 1 week, 1 month and change from baseline

Time point	PG-13-R score, mean (s.d.)	Change in PG-13-R score from baseline, mean difference (s.d.)
Baseline	24.9 (8.2)	Not applicable
1 week	21.2 (7.0)	−3.7 (6.0)***
1 month	19.8 (6.8)	−5.0 (6.9)***

***P < 0.001 (via paired-sample *t*-test).

Confounders such as education level, location (New York City versus outside the city) and relationship of deceased (spouse or child versus other kinship) were identified based on their significant bivariate associations with PG-13-R scores and code frequency (Table 3).

**Table 3:** Spearman correlations between participant characteristics and outcomes, grief severity (Prolonged Grief Disorder-Revised scale, PG-13-R) and code frequency

Outcome	Education		Location (New York City)		Relationship of deceased (spouse or child)	
	*r*	*P*	*r*	*P*	*r*	*P*
PG-13-R at baseline	−0.264	0.010*	0.118	0.260	0.209	0.041*
PG-13-R at 1 week	−0.182	0.080	−0.001	0.994	0.232	0.023*
PG-13-R at 1 month	−0.073	0.488	−0.031	0.768	0.310	0.002**
Change in PG-13-R, baseline to 1 week	0.144	0.167	−0.223	0.032*	0.023	0.826
Change in PG-13-R, baseline to 1 month	0.241	0.020*	−0.159	0.127	0.081	0.432
Change in PG-13-R, 1 week to 1 month	0.187	0.072	−0.008	0.939	0.086	0.407
Experiences with the deceased (positive)	−0.219	0.036*	0.212	0.043*	−0.029	0.784
Experiences with the deceased (negative)	0.145	0.168	−0.084	0.428	0.041	0.692
Experiences with the deceased (neutral/unclear)	−0.059	0.573	0.028	0.791	0.050	0.633
Experiences with the deceased (all valences)	−0.136	0.195	0.169	0.108	−0.018	0.865
Traits of the deceased (positive)	0.011	0.913	−0.008	0.937	−0.160	0.121
Traits of the deceased (negative)	0.226	0.030*	−0.015	0.889	−0.231	0.024*
Traits of the deceased (all valences)	0.081	0.444	0.025	0.810	−0.197	0.056
Death circumstances	−0.050	0.636	0.009	0.936	0.005	0.965
Regret and guilt	0.065	0.537	−0.083	0.431	0.042	0.689
Bitterness	0.174	0.098	0.123	0.243	0.035	0.738

*P < 0.05; **P < 0.01.

### Correlational results: associations between reminiscence variables and PGD symptom severity scores

We examined ten variables of reminiscence, derived from five thematic codes, with the emotional valences of codes treated as separate variables: experiences with the deceased (positive, negative, neutral/unclear and all valences); traits of the deceased (positive, negative and all valences); death circumstances; regret and guilt; and bitterness. We adjusted estimates for correlations between codes and PG-13-R scores to control for the total number of journal entries per participant and potential confounders identified above. Table 4 shows significant negative correlations between traits (negative) and PG-13-R scores at each time point: baseline (*r* = −0.302, *P* = 0.004), 1 week (*r* = −0.276, *P* = 0.008) and 1 month (*r* = −0.218, *P* = 0.037). There was a significant negative correlation between experiences (all valences) and PG-13-R score at baseline (*r* = −0.211, *P* = 0.044), but not at 1 week or 1 month. Negative correlations between experiences (neutral/unclear) and change in PG-13-R scores were found from 1 week to 1 month (*r* = −0.292, *P* = 0.005), and between regret and guilt and change in PG-13-R score from 1 week to 1 month (*r* = −0.219, *P* = 0.036).

**Table 4:** Spearman partial correlations between grief severity (Prolonged Grief Disorder-Revised scale, PG-13-R, scores) and code frequency

Code	PG-13-R at baseline		PG-13-R at 1 week		PG-13-R at 1 month		Change in PG-13-R, baseline to 1 week		Change in PG-13-R, baseline to 1 month		Change in PG-13-R, 1 week to 1 month	
	*r*	*P*	*r*	*P*	*r*	*P*	*r*	*P*	*r*	*P*	*r*	*P*
Experiences with the deceased (neutral/unclear)	−0.064	0.542	−0.026	0.806	−0.186	0.076	0.014	0.895	−0.114	0.281	−0.292	0.005**
Experiences with the deceased (all valences)	−0.211	0.044*	−0.122	0.245	−0.153	0.146	0.106	0.315	0.050	0.635	−0.093	0.377
Traits of the deceased (negative)	−0.302	0.004**	−0.276	0.008**	−0.218	0.037*	0.088	0.404	0.194	0.064	0.149	0.155
Regret and guilt	−0.017	0.870	0.004	0.970	−0.098	0.355	−0.020	0.851	−0.139	0.188	−0.219	0.036*

All correlations control for total number of journal entries; correlations also control for identified confounders: participant education level, living in New York City and relationship of deceased being a spouse or child.

*P < 0.05; **P < 0.01.

## Discussion

Remarkably, results demonstrated significant declines in PGD symptom severity long after the death of a significant other and after only 1 week of journalling in the LMH website, with effects sustained at 1-month follow-up. Given that the mean time since loss was 4 years and 9 months, these findings suggest that online journalling may have a therapeutic effect in reducing grief intensity, even some years after the death. Although the LMH was not developed intentionally to be psychotherapeutic, it nevertheless elicited narrative reflections on loss-related memories and emotions, and these reminiscences appear to have had some therapeutic benefits for those engaged in the journal. Consistent with other grief studies,^15,28,29^ we demonstrated that online daily writing provides a forum in which bereaved individuals can confront memories of the loss. Prompting bereaved individuals to confront grief-related thoughts and feelings was not shown to worsen PGD symptoms; rather, online journalling appeared to facilitate bereavement adjustment, suggesting promising new directions for the development of widely accessible grief therapies.

### Associations between reminiscence and PGD symptom severity

Results indicated that greater PGD symptom severity at baseline was associated with less reflection on past experiences and negative traits of the deceased in the LMH. Reflection on negative traits of the deceased was also associated with lower grief severity following LMH engagement. Findings suggest that LMH users who did not reflect on memories of the deceased – specifically past experiences or negative traits – had more severe PGD symptoms, aligning with the hypothesis that avoiding specific memories results in worse bereavement outcomes.^4-6^ Thus, results complement prior grief research showing that reflection on memories, even negative or emotionally distressing ones, facilitates bereavement adjustment when the goal is to make meaning of the loss.^19,20,29,30^ Overall, our findings lend support to the potential efficacy of guided online reflection to process grief and reduce symptoms of PGD (i.e. intense yearning, disbelief, numbness, pain and difficulty in moving on with life).

Analyses revealed an unexpected significant association between expressions of regret and guilt and reduction in PGD symptom severity 1 month after the first day of journal writing, contrary to our initial hypothesis that ruminating on one’s own distressing emotions in grief would confer worse bereavement outcomes. A possible explanation is that the LMH was a productive space for bereaved individuals to confront challenging emotions through expressive writing – the act of writing about deep, emotional experiences – which is shown to have psychotherapeutic outcomes.^31^ This aligns with contemporary grief models, which predict that periodically confronting grief-related emotions is integral to coping with grief,^17^ whereas an inability, or lack of opportunity, to share grief-related emotions and experiences characterises an isolating, persistent liminal state of grief that impedes adjustment.^32^ The LMH offered an alternative to sharing uncomfortable emotions with therapists, family or friends and perhaps provided an outlet for those without strong social support. Regarding expressions of guilt specifically, it is theorised that guilt can be psychologically adaptive if it promotes empathy for others and reparative changes in behaviour.^33^ However, specific forms of guilt may inhibit bereavement adjustment,^34^ particularly if it perpetuates rumination on counterfactual scenarios (i.e. preventing the death).^4^ It is possible that expressing guilt through online journalling promoted clarity through reflection rather than further rumination or circular thinking; however, our analysis did not index different forms of guilt, so further research is needed to unpack these results.

We also found a significant association between reflection on past experiences with neutral valence and reduction in PGD symptom severity from 1 week to 1 month. The ability to reflect on loss-related memories with less emotional bias (overly positive or overly negative) may be characteristic of grief adaptation and could be a sign of emotion regulation. PGD is characterised by symptoms of emotional pain and numbness, and research indicates that emotion regulation difficulties contribute to PGD symptoms.^2,35–37^ Similar studies have shown that reminiscence therapy – a psychosocial intervention delivered primarily to older adults and those at the end of life – reduces depression and anxiety symptoms and improves cognitive emotion regulation.^38–40^ Our findings might similarly represent a link between reminiscence and improved emotion regulation in grief.

Our results highlight how reminiscence and expressive writing may facilitate bereavement adjustment. Specifically, reflection on specific memories of the deceased and expression of regret and guilt were associated with low PGD symptom severity and improvement over time. Our study emphasises an important distinction: reflection on negative memories and emotions is not necessarily maladaptive and should not be conflated with grief rumination. Rather, confronting negative memories and working through distressing emotions probably paves the way for coping with grief. Our study advocates for narrative disclosure as a key component of bereavement support. The LMH may serve as a model for the development of more accessible online bereavement resources that centre reminiscence and expressive writing.

### Strengths and limitations

This study is novel in its application of mixed methods. Our collection of qualitative and quantitative data had the benefits of ease, accessibility and anonymity in using an online journal bereavement resource. We used a validated measure of grief symptom severity to explore any changes over the course of engagement in the LMH, and specifically the type of content entered. This was facilitated using a robust team approach to thematic analysis, which allowed us to identify and measure distinct patterns in LMH journals and any correlations with grief severity. The validity of our thematic analysis was supported by acceptable interrater reliability for most codes (Supplementary Table 3). However, thematic analysis of digital free text is limited, lacking the opportunity to ask follow-up or clarifying questions as in a semi-structured interview.

Another limitation of this study is the absence of a control group. The LMH journal was not designed specifically as a therapeutic intervention, and thus we refrain from drawing conclusions about its potential therapeutic benefits. Comparing an online expressive writing intervention with structured reflective prompts to a control condition lacking targeted guidance would enable a more rigorous assessment of reminiscence as a mechanism for bereavement adjustment. Furthermore, our study offers limited observation of PGD among LMH users, because most participants did not meet the diagnostic criteria for PGD based on either grief severity or the duration of time since the loss. Our sample captures bereavement experiences in the community, and grief generally varies in both severity and duration. Notably, the symptoms of grief (e.g. yearning for the deceased) were present in all subjects, as indicated by their responses to the PG-13-R, even if they did not meet the diagnostic criteria for PGD. The sample also encompasses a wide range of time since loss, from 2 months to 38 years, but is skewed towards those more recently bereaved, with a mean of approximately 5 years and a median of 2 years. This broad range introduces heterogeneity in grief trajectories, because participants may be at varying points in their mourning process. As a result, the study is limited in its ability to tailor recommendations to individuals experiencing prolonged grief. Future trials should evaluate a more targeted version of the LMH against a control intervention among individuals meeting diagnostic criteria for PGD.

Study participants were self-selected, predominantly White, female and highly educated, limiting study generalisability. In addition, 41 of the 137 participants withdrew from the study or were lost to follow-up. Regarding baseline characteristics, this group was older on average and had a higher percentage of Black and Hispanic/Latino participants than the group who completed the study. Further analysis may be warranted to evaluate the potential differential impact and acceptability of online bereavement interventions across age and race. It is possible that participants withdrew from the study due to time constraints and the level of effort required. This study required active participation in LMH writing for 20 min per day for 7 consecutive days. While more subjects might have completed the study if it had required less activity, this amount of engagement was associated with significant reductions in grief symptom severity, arguing in favour of this effort level. Future research should consider evaluation of the effects associated with lower ‘doses’ (i.e. time commitments).

Notwithstanding these limitations, this mixed-methods study of journal entries from the LMH bereavement website demonstrated that online journal writing exercises show promise in facilitating bereavement adjustment in a community-based, non-clinical sample. This study underscores the potential therapeutic benefits of reminiscence and expressive writing activities for bereaved adults. Future research may adapt the LMH into an exposure-based therapeutic intervention with more guided prompts for reflecting on memories of the deceased and processing emotional reactions to the loss.

## Supporting information

Lee et al. supplementary material Lee et al. supplementary material

## Data Availability

The data that support the findings of this study are available from the corresponding author, H.G.P., on reasonable request. The data are not publicly available due to privacy/ethical restrictions.

## Supplementary material

The supplementary material is available online at https://doi.org/10.1192/bjo.2025.10882

## References

[1] Shear MK. Grief and mourning gone awry: pathway and course of complicated grief. Dialog Clin Neurosci 2012; 14: 119–28. 10.31887/DCNS.2012.14.2/mshear PMC3384440 22754284

[2] Prigerson HG , Boelen PA , Xu J , Smith KV , Maciejewski PK. Validation of the new DSM-5-TR criteria for prolonged grief disorder and the PG-13-revised (PG-13-R) scale. World Psychiatry 2021; 20: 96–106. 33432758 10.1002/wps.20823 PMC7801836

[3] Prigerson HG , Singer J , Killikelly C. Prolonged grief disorder: addressing misconceptions with evidence. Am J Geriatr Psychiatry 2024; 32: 527–34. 38001019 10.1016/j.jagp.2023.10.020 PMC12210328

[4] Eisma MC , Schut HA , Stroebe MS , Boelen PA , van den Bout J , Stroebe W. Adaptive and maladaptive rumination after loss: a three-wave longitudinal study. Br J Clin Psychol 2015; 54: 163–80. 25229192 10.1111/bjc.12067

[5] Milman E , Neimeyer RA , Fitzpatrick M , MacKinnon CJ , Muis KR , Cohen SR. Rumination moderates the role of meaning in the development of prolonged grief symptomatology. J Clin Psychol 2019; 75: 1047–65. 30801707 10.1002/jclp.22751

[6] Eisma MC , Lang TA , Boelen PA. How thinking hurts: rumination, worry, and avoidance processes in adjustment to bereavement. Clin Psychol Psychother 2020; 27: 548–58. 32103569 10.1002/cpp.2440 PMC7497101

[7] Bryant RA , Kenny L , Joscelyne A , Rawson N , Maccallum F , Cahill C , et al. Treating prolonged grief disorder. JAMA Psychiatry 2014; 71: 1332–9. 25338187 10.1001/jamapsychiatry.2014.1600

[8] Bryant RA , Azevedo S , Yadav S , Cahill C , Kenny L , Maccallum F , et al. Cognitive behavior therapy vs mindfulness in treatment of prolonged grief disorder: a randomized clinical trial. JAMA Psychiatry 2024; 81: 646–54. 38656428 10.1001/jamapsychiatry.2024.0432 PMC11044011

[9] Shear MK , Reynolds CF , Simon NM , Zisook S , Wang Y , Mauro C , et al. Optimizing treatment of complicated grief: a randomized clinical trial. JAMA Psychiatry 2016; 73: 685–94. 27276373 10.1001/jamapsychiatry.2016.0892 PMC5735848

[10] Rosner R , Pfoh G , Kotoucova M , Hagl M. Efficacy of an outpatient treatment for prolonged grief disorder: a randomized controlled clinical trial. J Affect Disord 2014; 167: 56–63. 25082115 10.1016/j.jad.2014.05.035

[11] Webster JD. The reminiscence functions scale: a replication. Int J Aging Hum Dev 1997; 44: 137–48. 9169316 10.2190/AD4D-813D-F5XN-W07G

[12] Harris CB , Rasmussen AS , Berntsen D. The functions of autobiographical memory: an integrative approach. Memory 2014; 22: 559–81. 23808866 10.1080/09658211.2013.806555

[13] Eisma MC , Stroebe MS , Schut HAW , Van Den Bout J , Boelen PA , Stroebe W. Development and psychometric evaluation of the Utrecht Grief Rumination Scale. J Psychopathol Behav 2014; 36: 165–76.

[14] Wolf T , Strack V , Bluck S. Adaptive and harmful autobiographical remembering after the loss of a loved one. Aging Ment Health 2023; 27: 408–16. 34781789 10.1080/13607863.2021.2003299

[15] Eisma MC , Franzen M , Paauw M , Bleeker A , Rot M. Rumination, worry and negative and positive affect in prolonged grief: a daily diary study. Clin Psychol Psychother 2022; 29: 299–312. 34170063 10.1002/cpp.2635 PMC9291980

[16] Stroebe M , Boelen PA , van den Hout M , Stroebe W , Salemink E , van den Bout J. Ruminative coping as avoidance: a reinterpretation of its function in adjustment to bereavement. Eur Arch Psychiatry Clin Neurosci 2007; 257: 462–72. 17629726 10.1007/s00406-007-0746-y

[17] Stroebe M , Schut H. The dual process model of coping with bereavement: rationale and description. Death Stud 1999; 23: 197–224. 10848151 10.1080/074811899201046

[18] Boelen PA , van den Hout MA , van den Bout J. A cognitive–behavioral conceptualization of complicated grief. Clin Psychol Sci Pract 2006; 13: 109–28.

[19] Neimeyer RA , Baldwin SA , Gillies J. Continuing bonds and reconstructing meaning: mitigating complications in bereavement. Death Stud 2006; 30: 715–38. 16972369 10.1080/07481180600848322

[20] Neimeyer RA , Sands DC. Meaning reconstruction in bereavement: from principles to practice. In Grief and Bereavement in Contemporary Society: Bridging Research and Practice (eds RA Neimeyer, DL Harris, HR Winokuer, G Thornton): 9–22. Routledge, 2011.

[21] Gillies J , Neimeyer R , Milman E. The meaning of loss codebook: construction of a system for analyzing meanings made in bereavement. Death Stud 2014; 38: 207–16. 24524583 10.1080/07481187.2013.829367

[22] She W-J , Siriaraya P , Ang CS , Prigerson HG. Living memory home: understanding continuing bond in the digital age through backstage grieving. *Proceedings of the 2021 CHI Conference on Human Factors in Computing Systems (Yokohama, Japan, 8–13 May 2021)*. Association for Computing Machinery, 2021.

[23] Bailey L , Bell J , Kennedy D. Continuing social presence of the dead: exploring suicide bereavement through online memorialisation. New Rev Hypermedia Multimedia 2015; 21: 72–86.

[24] Irwin MD. Mourning 2.0—Continuing bonds between the living and the dead on facebook. OMEGA (Westport) 2015; 72: 119–50. 27132379 10.1177/0030222815574830

[25] Posner K , Brown GK , Stanley B , Brent DA , Yershova KV , Oquendo MA , et al. The Columbia–Suicide Severity Rating Scale: initial validity and internal consistency findings from three multisite studies with adolescents and adults. Am J Psychiatry 2011; 168: 1266–77. 22193671 10.1176/appi.ajp.2011.10111704 PMC3893686

[26] Maciejewski PK , Maercker A , Boelen PA , Prigerson HG. ‘Prolonged grief disorder’ and ‘persistent complex bereavement disorder’, but not ‘complicated grief’, are one and the same diagnostic entity: an analysis of data from the Yale Bereavement Study. World Psychiatry 2016; 15: 266–75. 27717273 10.1002/wps.20348 PMC5032512

[27] Neimeyer RA. Correspondence with the Deceased. Techniques of Grief Therapy: Assessment and Intervention 1st ed. Routledge, 2015.

[28] Eisma MC , Boelen PA , van den Bout J , Stroebe W , Schut HAW , Lancee J , et al. Internet-based exposure and behavioral activation for complicated grief and rumination: a randomized controlled trial. Behav Ther 2015; 46: 729–48. 26520217 10.1016/j.beth.2015.05.007

[29] Lichtenthal WG , Cruess DG. Effects of directed written disclosure on grief and distress symptoms among bereaved individuals. Death Stud 2010; 34: 475–99. 24482856 10.1080/07481187.2010.483332 PMC3909885

[30] Quayle K , Jones P , Di Simplicio M , Kamboj S , Pitman A. Exploring the phenomenon of intrusive mental imagery after suicide bereavement: a qualitative interview study in a British sample. PLoS One 2023; 18: e0284897. 37590210 10.1371/journal.pone.0284897 PMC10434947

[31] Pennebaker JW. Writing about emotional experiences as a therapeutic process. Psychol Sci 1997; 8: 162–6.

[32] Bristowe K , Timmins L , Pitman A , Braybrook D , Marshall S , Johnson K , et al. Between loss and restoration: the role of liminality in advancing theories of grief and bereavement. Soc Sci Med 2024; 344: 116616. 38310729 10.1016/j.socscimed.2024.116616

[33] Tangney JP , Stuewig J , Mashek DJ. Moral emotions and moral behavior. Annu Rev Psychol 2007; 58: 345–72. 16953797 10.1146/annurev.psych.56.091103.070145 PMC3083636

[34] LeBlanc NJ , Toner ER , O’Day EB , Moore CW , Marques L , Robinaugh DJ , et al. Shame, guilt, and pride after loss: exploring the relationship between moral emotions and psychopathology in bereaved adults. J Affect Disord 2020; 263: 405–12. 31969271 10.1016/j.jad.2019.11.164 PMC7307182

[35] Burton CL , Yan OH , Pat-Horenczyk R , Chan IS , Ho S , Bonanno GA. Coping flexibility and complicated grief: a comparison of American and Chinese samples. Depress Anxiety 2012; 29: 16–22. 21898713 10.1002/da.20888 PMC3242921

[36] Gupta S , Bonanno GA. Complicated grief and deficits in emotional expressive flexibility. J Abnorm Psychol 2011; 120: 635–43. 21766973 10.1037/a0023541

[37] Gegieckaite G , Kazlauskas E. Do emotion regulation difficulties mediate the association between neuroticism, insecure attachment, and prolonged grief? Death Stud 2022; 46: 911–9. 32628562 10.1080/07481187.2020.1788667

[38] Bozkurt C , Erbay-Dalli O , Yildirim Y. The effectiveness of reminiscence therapy on anxiety, depression, and quality of life in adult cancer patients: a systematic review and meta-analysis. Support Care Cancer 2024; 32: 728. 39402338 10.1007/s00520-024-08920-6

[39] Ramadan Abdel-Aziz H , AbdElkhalek Ahmed HA. The effect of group reminiscence therapy on self-esteem and emotional well-being of older adults. Cent Eur J Nurs Midwif 2021; 12: 513–20.

[40] Wu D , Chen T , Yang H , Gong Q , Hu X. Verbal responses, depressive symptoms, reminiscence functions and cognitive emotion regulation in older women receiving individual reminiscence therapy. J Clin Nurs 2018; 27: 2609–19. 29119637 10.1111/jocn.14156

