# Supplementary material for: Reminiscence and grief resolution through online journalling: mixed-methods analysis of the Living Memory Home bereavement cohort
Source: BJPsych Open. 2025 Oct 28;11(6):e259. doi: 10.1192/bjo.2025.10882 (PMC12569618; doi:10.1192/bjo.2025.10882)
Supplement: Lee et al. supplementary material [file S205647242510882Xsup001.docx]

**Supplementary Material**

**S1 Appendix.** Detailed Methodology for Thematic Analysis

**S2 Appendix.** Living Memory Home Writing Prompts

**S1 Table.** Codebook for the Living Memory Home Journal Entries

**S2 Table.** Frequency of Codes in the Living Memory Home Journal Entries

**S3 Table.** Final Interrater Reliability

**References**

**S1 Appendix.** Detailed Methodology for Thematic Analysis

**Data Extraction**

All text entries were deidentified and extracted from the website for analysis, with sequential entries grouped at the individual level. The dataset included the participant-selected writing prompts and corresponding free text entries in chronological order for each participant. Deidentified data were managed and organized using Microsoft Excel. In total, the analysis included 2,390 journal entries from 96 participants.

**Coding Team**

The final coding was conducted by six research members: a licensed social worker with a master’s degree, two medical students, and three college graduates with majors in psychology. A seventh member, a developmental psychologist, was present during codebook development but did not participate in the final coding.

**Codebook Development**

The thematic analysis followed Braun & Clarke’s 6-step framework.^3^ Coding members (above) familiarized themselves with the data by independently reviewing and taking notes on a random sample of 300 text entries, which represented 15% of the total available entries at that time. The team anticipated that a 15% sample would be sufficient for data familiarization and initial observations, given the large number of journal entries and depth of writing in the LMH. The team discussed initial impressions and generated codes based on distinct observations in the data. The team drafted a working codebook, which included a list of all codes, organized by theme, with clear definitions and textual examples. The team’s expertise in psychology and grief-related research informed the conceptual meaning of the codes as well as the organization of codes into themes.

Codebook development followed and iterative induction-deduction process, repeated across multiple rounds of independent coding and consensus meetings. Coding rounds were conducted for the purposes of refining the codebook and assessing interrater reliability (IRR). In each coding round, coding members independently coded whole sets of journal entries from a random sample of 3 participants, summing to about 60-90 text entries per coding round. This sample size balanced both time efficiency and scope: the sample size was sufficient to capture the range of codes in our codebook, and manageable for members to independently code for bi-weekly consensus meetings. Following each coding round, a consensus meeting was held to compare coding across members, discuss coding discrepancies, clarify the conceptual meaning of codes, and revise the codebook by merging, deleting, or creating new, appropriate codes. Coding members took turns leading consensus meetings. In rare cases when consensus could not be reached by discussion alone, a vote was held to reach a final coding decision. To evaluate the psychometric properties of the codebook, we calculated IRR using Cohen’s kappa value, a common measure of IRR in thematic analyses,^4-6^ for each code for every coding round. A kappa value ≥ .60 for each code, for at least the top 75% of the most frequent codes, was considered an acceptable level of agreement.^7,8^ Coding rounds and consensus meetings continued until target IRR (as specified above) was achieved, and no further codebook revisions were required, indicating data saturation. We achieved acceptable agreement and data saturation after seven coding rounds, which is comparable to other thematic analyses with similar design.^4^ At this stage, the codebook was considered finalized.

**Coding Protocol**

Coding members were instructed to complete a first pass reading of a participant’s total entries in chronological order without coding. Then, on a second pass, codes were applied to each entry. Completing a first pass read-through helped the coder contextualize each text entry within the whole of a participant’s journal writing, improving coding accuracy and reliability. Regarding the level of interpretation, coding members were instructed to employ a semantic, or “surface-level,” approach to interpreting the text, as opposed to a deeper “latent” interpretation. Using this approach, coders observed the literal and descriptive meanings of the text but did not infer beyond the text or make assumptions about the writer. This approach was chosen to optimize coding reliability and accurately characterize the LMH journals.

Codes were applied to individual text entries, and multiple codes could be applied to a text entry. Thus, each code was counted as either present or absent for each entry. We conceptualized a text entry was a distinct unit of meaning. This aligned with our understanding of each text entry as a complete and contained thought pertaining to a particular writing prompt. If a text entry was incomplete or unreadable, then the code of “No Theme” was applied.

**Final Coding**

The full data set was coded using the final version of the codebook. Thus, all entries that were reviewed during codebook development were re-coded in the final coding of the full data set. The full dataset was distributed between three coding pairs (FO + MR, CLE + CLA, and SK + KP). Coding was conducted independently with random 25% overlap between coders in a coding pair for the calculation of final IRR for each code (S3 Table). The 25% overlap consisted of participants’ whole sets of journal entries. For instance, if a coding pair was assigned 32 participants, then a random sample of 8 participants was coded by both members for final IRR calculation, and the remaining 24 participants were evenly divided for independent coding. In other qualitative analyses of similar design, the size of overlap might range anywhere from 10% to 25% of the whole sample.^6,9,10^ We chose a relatively high percentage of overlap to obtain accurate IRR calculations. Consensus meetings were held to resolve all coding discrepancies for double-coded entries, for the purpose of attaining a final code count for each entry.

**Code Frequency**

Final code counts were used as variables for statistical analysis. For each participant, we counted the frequency of codes as the sum of individual text entries expressing a given code. For instance, if a participant wrote 30 total journal entries, and 10 of those entries were coded “Death Circumstances,” then the code frequency for “Death Circumstances” would equal 10 for that participant. We decided that the total number of entries, as opposed to word count or character count, was the most appropriate measure of code frequency. This measure aligned with our conceptualization of each entry as a complete thought and distinct unit of meaning in our coding protocol. Code counts were calculated for each participant, for each code. The total frequency of codes, summed across all study participants, is provided in S2 Table.

**S2 Appendix**. Living Memory Home Writing Prompts

1. What I have always wanted to tell you is...
2. Thoughts of pride or shame about you that I want to share are...
3. Thoughts of pride or shame about me that I want to share are...
4. One of my most treasured memories of you is...
5. One of my most frightening memories of you is...
6. One of my most frightening memories of my life is...
7. What you never understood was...
8. Only you understood that I...
9. Without you, I feel others...
10. What I want you to know about me is...
11. Without you, I feel like...
12. I miss when we...
13. What I now realize is...
14. Without you I no longer...
15. Now that you’re gone I feel...
16. The one question I have wanted to ask is...
17. I regret that we never...
18. When I miss you I...
19. I want to keep you in my life by...
20. Because I miss you I...
21. To honor your memory I...
22. What's on your mind today?
23. If [deceased] could say something to me now, it would likely be...
24. The way I think [deceased] would wish to be remembered would be...
25. One thing I learned from [deceased] that leads to living well, being happy, and honoring the values I hold is...
26. Some qualities of [deceased] that I wish to emulate in my own life are...
27. l think of you the most when I...
28. One thing that has surprised me is...
29. As I think about our relationship, what feels most important is...
30. I feel that I am needing ...
31. One thing I still wonder about is...
32. One thing I am struggling to accept ... (one of the hardest things for me to let go of right now is...)
33. I will continue to heal by...
34. I feel the most at peace when I ...
35. One thing I’ve noticed is...
36. Your greatest gift was...
37. My first memory of you...
38. One memory I never want to forget is...
39. The most touching thing about you I remember is...
40. Time together felt like...
41. You found strength to overcome the inevitable difficulties in life by...
42. Something you would say when I was having a hard time was...
43. The place where I think of you the most...(or where I go to think of you... )
44. [deceased], I feel your presence in the things you left behind...

**S1 Table.** Codebook for the Living Memory Home Journal Entries

| **Code** | **Definition** | **Textual Examples** |
| --- | --- | --- |
| **Theme: Reflection** | | |
| Experiences with the Deceased | Describing specific, lived events involving the deceased. This may include events experienced by the writer (participant) or others. Further coded by emotional valence: Positive, Negative, or Neutral/Unclear. | Positive: “*One memory I never want to forget is*… going with you to parrots of the world and watching you greet each of the parrots individually. You enjoyed that so much and it was such a treat to watch you.”  Negative: “My most frightening memory of you is when you had a seizure outside while I was on the balcony. All I saw was your friend screaming for help and you were face down on the ground with blood splattered all across the ground.”  Neutral/Unclear: “*My first memory of you*… You in a white tank top at [location]." |
| Traits of the Deceased | Describing specific characteristics or values of the deceased. Further coded by emotional valence: Positive or Negative. | Positive: “You were so expressive and so alive. You seemed so comfortable in yourself around us.”    Negative: “...everything you did was always about you first. Rarely did you think about how things affected me...only to consistently apologize for your selfish behavior” |
| Daily Reporting | Giving an account of daily, current activities and happenings. Frequently coded in response to the "What's on your mind today?" prompt. Further coded by emotional valence: Positive, Negative, or Neutral/Unclear. If both positive and negative elements exist in the same entry, code as “Neutral/Unclear.” | Positive: “The stars were out tonight and I looked up to a very clear sky which is unheard of in the city.”  Negative: “It's a sad day. My dad is on my mind a lot. I was busy at work but something is still missing. I feel an emptiness today.”  Neutral/Unclear: “I don't really have much on my mind today- I sometimes go to a place of numbness and it works." |
| Outlook on Life & Personal Beliefs | Discussing questions and attitudes about the future or human existence in general. Discussing personal beliefs or philosophies that could also include spiritual and/or religious values. | "I will always love you. I believe that life does not end when the heart stops beating and I know we will see one another again.”    “Why do good people need to suffer? Why is the world not fair? Why can't we have sustainable goodness?" |
| Death Circumstances | Describing specific memories of the deceased person’s end-of-life, the death itself, or the immediate aftermath/impact of the death. Consider double coding with “Experiences with the Deceased" when pre-death circumstances are mentioned. | “Seeing you for one of the last times, and laying next to you in the hospice bed when you were decimated from the cancer and the chemo.”  “This is the house I brought him home to and the house he lived in when he died. Some people told me to move out.....leave after he died knowing he died here.” |
| Unresolved Questions | Describing things left unsaid, unanswered or unresolved, a lack of closure with the deceased. Does NOT apply to general, existential questions unrelated to the deceased, such as “Why do some people die and others do not?” Frequently coded in response to “The one question I have wanted to ask is...” prompt. | “What did you want me to do? What kind of woman do you want me to be? What should I do at times?”  “you were hiding much more pain than I knew. Why couldn't you share it with me. Did you try and I didn't see it?” |

| **Theme: Continued Bonds** | | |
| --- | --- | --- |
| Referential & Legacy | Feeling connected to the deceased through a receptive experience (i.e. through symbols such as personal belongings, places, photographs, etc.) or through an expressive act that maintains a legacy in their memory (e.g. act of services, emulating traits of the deceased, generativity). | Receptive: "I think of you most when I am running along the canal. The airplanes fly over which I know was one of your favorite things. I also see all of nature and the beauty in it. There are always cardinals on my morning runs and even though I am not religious- I hope that someway nature is communicating.”  Receptive: “I feel your presence when I see your pictures. Your jewelry is still with me."  Expressive: "Even though I have lost so much, I have a lot of gratitude and try to keep your spirit alive by being happy, engaged with the world, and reliable.”  Expressive: “I wonder if you understood what an impact you had on not only your family, but the community around you. You were so involved in the university, local and state communities, especially with swimming and diving-- and that legacy is still living on today." |
| Identification (Between deceased and self) | Observing similar traits/qualities/influences between oneself and the deceased. | "Someone had brought up that I have a lot of his tastes, like what he likes to drink since I turn 21 this week. And I was telling them the drinks that I like, which were just like Dad's. I think also the small things: foods he liked, how he dressed, the way he greeted people, his warm smile and demeanor. And his values and intensity. He always fought for what was right and just, and I've been able to be the same way.”    “You were amazing in your ability to talk to people and just kind of "get them". I like to think that it was one of my greatest strengths and I think I get that from you. Thank you.” |
| Identification (Between deceased and others) | Observing similar traits/qualities/influences between others and the deceased. | “I see you in the twinkle of mummy's eyes. . . Mummy is the reflection of you.”    “I feel your presence in my sister. She has some of your traits. A joy of entertaining. She likes to garden. She has this traditional sensibility that no Gen-Xer should have." |
| Imagined Perspective of the Deceased | Using the voice of the deceased to consider or describe what they would think, feel, do, or say in a given situation occurring in the present day. | “[name] had many sayings...right now he would probably say "Pleasure has no price". The dogs daycare costs went up but they love it so much that I will make it work.”    “would probably be spinning in his kitchen right now and he would just tell me to come sit and put his finger on my shoulder." |
| Admiration & Pride | Describing the deceased as an aspiration or role model, and/or feeling proud of the deceased. Key terms may include: “aspire,” “proud,” “role model”. Merely describing the deceased in a positive light is NOT sufficient for this code (see *Traits of the Deceased*). | “I admire and look up to you. I want to be as good as of a person as you were. It's hard in today's world to be that person but I also think those people are needed.”    “Proud that you were my mother - you were so authentic as a person.” |
| **Theme: Psychosocial Adjustment** | | |
| Regret & Guilt | A feeling of repentance or remorse regarding an unfavorable outcome; wishing different decisions/actions had been taken in the past. Must be in the context of the deceased and self-directed. | “...We didn't talk much about feelings when you were alive and that is one thing I regret not doing more of.”  “I wish I could go back and tell you what I know to say now. I wish I could have sat with you in your sadness.”    “I feel guilty because you're not here to enjoy it. You loved life so much!!” |
| Yearning & Disbelief | Actively missing or wishing for the deceased; a strong feeling of need or desire for the deceased. A sense of struggling to accept the reality of the loss. Key terms may include: “miss you,” “wish you were here.” | “I miss cooking, I miss the holidays, I miss our birthdays. I miss garbage night. I miss dr appts. I miss your cooking. So much to miss it can't be listed in paragraphs. I miss my mother and my best friend. I, I, I. I miss you.”    “It's been over a year and it is still so hard to accept that you are no longer with us.” |
| Internal Disruption | Observing a change in one’s sense of identity or mental health connected to the loss of the deceased (i.e. feeling empty or a part of oneself is missing). | “Empty- like a part of me is missing. Many times I just feel lost. (Not sure who I am anymore).”  “Without you, I feel like . . . I feel a bit rootless and ungrounded. I feel more insecure and anxious. I feel like I'm searching.” |
| External Disruption | Observing a change in one’s life, reality, or routine connected to the loss of the deceased. This includes changes experienced in relationships, family dynamics, and other life disturbances resulting from the loss of the deceased. | “I feel others are not connected like they used to be. Our family members don't talk to each other. You were the binder.”  “Without you I no longer have a parent who loves me unconditionally. … I never got to experience what that is like to have a parent while becoming a parent.” |
| Difficulty Tolerating Memories | Explicit statements that attempting to recall memories of the deceased and the loss causes distress. | “She was my smallest baby and it's very painful even now twenty years later just remembering what life was like for me, for us all then.” |
| Difficulty Recalling Memories | A stated inability or struggle to recall memories of the deceased and/or a fear of forgetting memories of the decease. | “After last night struggling to find some happy things about our relationship, other than sex, see above, and still cannot think of anything. Yes, searching my brain and still coming up blank.”  “I can't remember ever feeling sad around you. I can't think of how you consoled me. It must have happened in the past but I can't remember.” |
| Bitterness | Expressing negative thoughts and feelings (e.g. anger, resentment, disappointment, etc.) towards the deceased, other people, or one’s circumstances. Often involves use of hyperbole, or exaggerated language. Does NOT have to be directed toward the deceased. | “...Everything you did was always about you first. Rarely did you think about how things affected me...only to consistently apologize for your selfish behavior”  “Papa was cold he was unable to cry. I was shocked. You have it so bad he said to me, turning off all his feelings. He never hugged me, never gave a word of consolation. I suffered it all alone.” |
| Pity & Empathy | Describing a feeling of sorrow and compassion toward the deceased for their suffering or misfortunes. | “I know you had end stage Alzheimer's Disease, and it was so hard to know what you were thinking or experiencing. We all felt so badly - it was heartbreaking actually- that they would not allow us to visit with you toward the last few weeks of your life because of the covid 19 pandemic. I hope you didn't think we abandoned you or gave up on you.”  “Your final days alive were awful and full of so much pain, I can still see you in that state. I wish you didn't have to go through that.” |
| Reminder Avoidance | Explicitly distracting oneself from or avoiding reminders (e.g. pictures, belongings, places, etc.) of the deceased and the loss. | "… brought home your clothes for us to donate and I couldn't even look at them”    “i feel very lonely and upset sometimes. i always try to distract myself by engaging in other activities " |
| Resilience | A sense of overcoming hardships and/or coping with a stated problem (e.g., grief, day-to-day struggles, etc.); finding strength in oneself or envisioning a path forward. This does not have to be related to the loss of the deceased. | “I embrace my grieving and give myself the time to feel it fully. I've been dulled by alcohol for the past three years and it's time to feel again. Self care helps with healing and I check myself if the self care is turning into selfishness. I volunteer in roles in which I have to actively listen. The less time I spend in my head the more time I give myself to live.”  “I was in tremendous crisis, but somehow I found strength to pull through.” |
| Mental Health | Explicitly describing one’s own mental health needs, concerns, or psychiatric history that are distinct from grief and unrelated to the loss. | “I need assistance for my family, and we are having mental health crises today as a result of the abuse we suffered.”  “I have also been a had a bit of underlying anxiety. I'm not sure how to feel completely relaxed and as always am not including daily practices.” |
| **No theme** | | |
| No theme | An entry that cannot be categorized with any of the above codes. An incomplete or incomprehensible entry. | “TTTT”  “*Only you understood that I*… am your sister.” |

**S2 Table.** Frequency of Codes in the Living Memory Home Journal Entries

| **Theme** | **Code** | **Frequency**  **(Number of entries)** |
| --- | --- | --- |
| Reflection | Traits of the Deceased (Positive) | 541 |
|  | Experiences with the Deceased (Positive) | 338 |
|  | Outlook on Life / Personal Beliefs | 310 |
|  | Death Circumstances | 241 |
|  | Daily Reporting (Negative) | 210 |
|  | Daily Reporting (Neutral/Unclear) | 188 |
|  | Unresolved Questions | 152 |
|  | Daily Reporting (Positive) | 151 |
|  | Traits of the Deceased (Negative) | 134 |
|  | Experiences with the Deceased (Negative) | 129 |
|  | Experiences with the Deceased (Neutral/Unclear) | 51 |
| Continued Bonds | Referential & Legacy | 570 |
|  | Imagined Perspective of the Deceased | 256 |
|  | Admiration & Pride | 113 |
|  | Identification (Between deceased and self) | 61 |
|  | Identification (Between deceased and others) | 23 |
| Psychosocial Adjustment | Yearning & Disbelief | 303 |
|  | Resilience | 242 |
|  | Internal Disruption | 195 |
|  | Regret & Guilt | 188 |
|  | External Disruption | 164 |
|  | Bitterness | 159 |
|  | Pity & Empathy | 123 |
|  | Mental Health | 112 |
|  | Difficulty Tolerating Memories | 54 |
|  | Reminder Avoidance | 37 |
|  | Difficulty Recalling Memories | 35 |
| No theme | No theme | 104 |

**S3 Table.** Final Interrater Reliability

| **Code** | **Coders FO + MR**  **(N = 191 entries)** | | **Coders CLE + CLA**  **(N = 170 entries)** | | **Coders SK + KP**  **(N = 148 entries)** | |
| --- | --- | --- | --- | --- | --- | --- |
|  | **Frequency (Number of entries)** | **Kappa value** | **Frequency (Number of entries)** | **Kappa value** | **Frequency (Number of entries)** | **Kappa value** |
| Experiences with the Deceased (Positive) | 18 | 0.77* | 24 | 0.72* | 18 | 0.63* |
| Experiences with the Deceased (Negative) | 16 | 0.64* | 3 | 0.50 | 9 | 0.76* |
| Experiences with the Deceased (Neutral/Unclear) | 5 | 0.43 | 2 | 0 | 7 | 0.35 |
| Traits of the Deceased (Positive) | 53 | 0.91* | 27 | 0.74* | 40 | 0.76* |
| Traits of the Deceased (Negative) | 11 | 0.69* | 4 | 0.80* | 3 | 0.66* |
| Daily Reporting (Positive) | 15 | 0.71* | 18 | 0.78* | 1 | 0 |
| Daily Reporting (Negative) | 18 | 0.72* | 14 | 0.88* | 12 | 0.64* |
| Daily Reporting (Neutral/Unclear) | 22 | 0.59 | 18 | 0.51 | 12 | 0.75* |
| Outlook on Life / Personal Beliefs | 27 | 0.59 | 34 | 0.56 | 23 | 0.64* |
| Death Circumstances | 29 | 0.71* | 12 | 0.65* | 21 | 0.60* |
| Unresolved Questions | 14 | 0.79* | 6 | 0.66* | 12 | 0.67* |
| Referential & Legacy | 48 | 0.85* | 43 | 0.70* | 40 | 0.67* |
| Identification (Between deceased and self) | 6 | 0.66* | 0 | - | 3 | 0.56 |
| Identification (Between deceased and others) | 5 | 0.32 | 3 | 1.00* | 1 | 0 |
| Imagined Perspective of the Deceased | 13 | 0.68* | 14 | 0.66* | 21 | 0.68* |
| Admiration & Pride | 7 | 0.48 | 4 | 0.56 | 7 | 0.47 |
| Regret & Guilt | 17 | 0.88* | 11 | 0.69* | 8 | 0.94* |
| Yearning & Disbelief | 25 | 0.83* | 24 | 0.87* | 23 | 0.78* |
| Internal Disruption | 9 | -0.02 | 23 | 0.65* | 19 | 0.65* |
| External Disruption | 12 | 0.57 | 19 | 0.61* | 7 | 0.65* |
| Difficulty Tolerating Memories | 4 | 0.86* | 1 | 1.00* | 5 | 0.59 |
| Difficulty Recalling Memories | 5 | 0.75* | 3 | 0.80* | 1 | 0 |
| Bitterness | 10 | 0.14 | 2 | -0.01 | 7 | 0.83* |
| Pity & Empathy | 13 | 0.75* | 6 | 0.39 | 5 | 0.49 |
| Reminder Avoidance | 2 | 0.67* | 3 | 0 | 2 | 0.66* |
| Resilience | 10 | 0.77* | 23 | 0.63* | 16 | 0.46 |
| Mental Health | 5 | 0.66* | 7 | 0.60* | 13 | 0.06 |
| No theme | 9 | 0.83* | 6 | 0.85* | 2 | 0 |

*Indicates acceptable agreement (kappa value ≥ .60)

**References**

1. She W-J, Siriaraya P, Ang CS, Prigerson HG. Living Memory Home: Understanding Continuing Bond in the Digital Age through Backstage Grieving. presented at: Proceedings of the 2021 CHI Conference on Human Factors in Computing Systems; 2021;

2. Neimeyer RA. Correspondence with the Deceased. *Techniques of Grief Therapy: Assessment and Intervention*. 1st ed. Routledge; 2015.

3. Braun V, Clarke V. Using thematic analysis in psychology. *Qualitative Research in Psychology*. 2006;3(2):77–101.

4. Hruschka DJ, Schwartz D, St.John DC, Picone-Decaro E, Jenkins RA, Carey JW. Reliability in Coding Open-Ended Data: Lessons Learned from HIV Behavioral Research. *Field Methods*. 2004;16(3):307-331. doi:10.1177/1525822x04266540

5. Burla L, Knierim B, Barth J, Liewald K, Duetz M, Abel T. From Text to Codings: Intercoder Reliability Assessment in Qualitative Content Analysis. *Nursing Research*. 2008;57(2):113-117. doi:10.1097/01.NNR.0000313482.33917.7d

6. MacPhail C, Khoza N, Abler L, Ranganathan M. Process guidelines for establishing Intercoder Reliability in qualitative studies. *Qualitative Research*. 2016;16(2):198-212. doi:10.1177/1468794115577012

7. Cohen J. A Coefficient of Agreement for Nominal Scales. *Educational and Psychological Measurement*. 1960;20:37 - 46.

8. McHugh ML. Interrater reliability: The kappa statistic. *Biochemia Medica*. 2012;22(3):276-82. doi:<https://doi.org/10.11613/BM.2012.031>

9. Campbell JL, Quincy C, Osserman J, Pedersen OK. Coding In-depth Semistructured Interviews. *Sociological Methods & Research*. 2013;42(3):294-320. doi:10.1177/0049124113500475

10. O’Connor C, Joffe H. Intercoder Reliability in Qualitative Research: Debates and Practical Guidelines. *International Journal of Qualitative Methods*. 2020;19doi:10.1177/1609406919899220
